# Medication Adherence in Cancer Patients: A Comprehensive Review

**DOI:** 10.7759/cureus.52721

**Published:** 2024-01-22

**Authors:** Reshma V, Arun M Chacko, Naseeha Abdulla, Maduram Annamalai, Venkataramana Kandi

**Affiliations:** 1 Pharmacology, Shri Sathya Sai Medical College and Research Institute (SSSMCRI), Chennai, IND; 2 Biochemistry, Sri Balaji Vidyapeeth, Puducherry, IND; 3 Biochemistry, Azeezia Institute of Medical Sciences and Research, Kollam, IND; 4 Pharmacology and Therapeutics, Kunhitharuvai Memorial Charitable Trust (KMCT) Medical College, Calicut, IND; 5 Clinical Microbiology, Prathima Institute of Medical Sciences, Karimnagar, IND

**Keywords:** risk factors, facilitators to medication adherence, barriers, therapeutic modalities, medication adherence, cancer

## Abstract

Cancer is a complex disease that can affect different parts of the body. The rates of cancer have shown an increasing trend in the past decade. A majority of cancers are detected late, therefore becoming untreatable and resulting in significant mortality. Additionally, the lack of awareness about cancers, their risk factors, diagnostic modalities, and preventive measures contributes to increased burden among people. Despite significant developments in the therapeutic and comprehensive management of cancers, the cause for concern is the lack of medication adherence. This is majorly attributed to the adverse effects of the medication, the cost of the drugs, and other reasons. This review comprehensively discusses various aspects of cancer medication adherence that include therapeutic modalities for treating cancers, factors influencing medication adherence, barriers, and facilitators to medication adherence.

## Introduction and background

Globally, cancer is the second leading cause of death after cardiovascular diseases. Cancer accounted for more than 10 million deaths in 2020. Over the next 20 years, it is anticipated that there will be 22 million incidences of the disease every year, up from 14 million in the year 2012 [1-3]. Moreover, there is an increasing trend of cancers caused by the human papillomavirus (HPV) and hepatitis viruses (hepatitis B virus and hepatitis C virus), especially among people residing in low- and low-middle-income countries such as India [1]. The International Agency for Research on Cancer (IARC), a research group headed by the World Health Organization (WHO), suggested cancer causes that include exposure to radiation, chemicals, toxins, and microbes [1]. According to the WHO, identifying risk factors, minimizing the risk, screening, early diagnosis, treatment, and palliative care are essential to reduce the burden of cancer [1]. With an anticipated 2.3 million new cases worldwide in 2020, breast cancer (BC) has surpassed lung cancer as the most frequently diagnosed malignancy [4]. The impact of the obesity epidemic and the mammography screening program, which preferentially finds slow-growing estrogen receptor (ER)-positive tumors, may be blamed for the rise in ER-positive breast cancer, according to the data that are currently available in several different nations [5]. Advanced technologies have resulted in new drug interventions, and these can extend life expectancy by reducing the progression of cancer and sometimes cure the disease, improving the patient's quality of life. Adherence to the treatment advice with adequate measures is a vital part of the cure of the disease [6]. This review comprehensively discusses various aspects of cancer medication adherence, such as therapeutic modalities for treating cancers, factors influencing medication adherence, barriers, and facilitators to medication adherence.

## Review

Cancer treatment modalities

Cancer treatment is complex and comprises approaches such as surgery, radiation, and chemotherapy. However, more advanced methodologies such as gene therapy, stem cell therapy (induced pluripotent stem cells, embryonic stem cells, hemopoietic stem cells, neuronal stem cells, and cancer stem cells), monoclonal antibodies, nano-based medicine, ablation (thermal ablation, cryoablation, and radiofrequency ablation), and chemodynamic and ferroptosis-based therapies, along with radiodynamic therapies, radionics, chitosan-based photodynamic therapy, targeted delivery-based therapy modalities using folic acid-conjugated photo-responsive polymeric particles, oncolytic virus-based therapy, and natural antioxidant therapies, have been explored in the cancer treatment. Some of these interventions are at different stages of development, and a few are already being approved and utilized for the treatment of various cancers [7-11]. The anticancer properties of thioredoxin-binding protein (TXNIP) have been investigated for its application to treat cancer [12]. Recently, polymer-directed enzyme prodrug therapy, elastin-like polypeptides, and plasmid deoxyribonucleic acid (DNA) have been suggested as newer treatment options for cancer [13-15].

**Figure 1:**
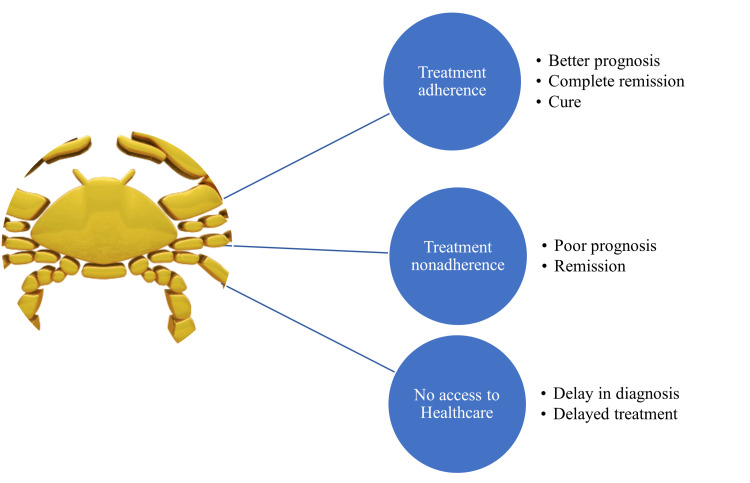
Pictorial representation of the cancer medication adherence factors and consequences Image credits: Venkataramana Kandi

Studies reported that poor therapeutic adherence is a significant issue among patients who were prescribed oral therapeutic agents for cancer. Understanding the multiple issues patients confront throughout the therapy is crucial for addressing the issue of medication nonadherence [16]. Treatment adherence could result in a better prognosis, complete remission, and cure for cancer. Alternatively, medication nonadherence leads to poor prognosis and the remission of cancer. Moreover, the lack of access to healthcare facilities causes delays in the diagnosis and treatment initiation. The consequences of medication adherence and nonadherence in cancer patients are depicted in Figure 1.

Anticancer therapeutic adherence

According to the World Health Organization (WHO), adherence is characterized by the behavior of the patient who abides by the physician's prescription of treatment and diet and lifestyle change recommendations. The WHO does not define nonadherence to anticancer therapy [17,18]. The implementation/medication possession ratio of more than 80% of the prescribed medicines and the persistence of the therapeutic regimen for the complete course, as recommended by the clinicians, are recognized as medication adherence. The discontinuation of the prescribed drug for more than 60-180 days may be considered nonadherence [19]. Even though anticancer agents are currently becoming increasingly available and have become accessible and affordable, there is still a massive concern over adherence to therapeutic regimens.

Few studies in the past have attempted to understand the factors for nonadherence to medications among cancer patients. However, most of these studies were plagued with bias, and therefore, medication adherence continues to be a significant hurdle in the management of cancer. Some factors that may affect medication adherence include the cost of the treatment, the complexity of the therapeutic regimens, drug-food interactions, drug-drug interactions, adverse effects, and patient-related factors such as forgetfulness and carelessness [20]. Recently, telemedicine, mobile applications, and electronic diaries such as a remote monitoring system to optimize the home management of oral anticancer therapies (ONCO-TreC) were suggested to improve the monitoring of patients regarding medication adherence [21].

Another major hurdle associated with poor medication adherence, especially among low-income countries, is the nonavailability of medical insurance. A study from Columbia by Sood et al. noticed that the odds of nonadherence to adjuvant hormone therapy (AHT) were considerably low among females who were medically insured [22].

In a study by Zhao et al. from the United States of America, females aged <40 years and ≥80 years were noted to have high nonadherence rates to anticancer therapeutic regimens. The high cost of the regimen, multiple drugs, depression, the presence of other comorbidities, and having undergone oophorectomy were factors that influenced nonadherence. This study also observed that despite the increase in the AHT initiation rates (90%), there was a low adherence (65%) to therapy [23]. Other factors that could influence adherence to an anticancer regimen include adverse effects of the drugs (sleep disturbance, hot flashes, joint pain, fatigue, and anxiety), social support, and effective drug-related communication between the provider and the patients [24,25].

Hormonal therapy and breast cancer

Adherence to cancer therapy assumes increased significance, especially in patients diagnosed with hormone receptor (HR)-positive breast cancer (BC). These patients are generally prescribed therapy for 5-10 years. Moreover, long-term adherence among disadvantaged populations belonging to low- and low-middle-income countries could result in increased mortality [26].

AHT is beneficial for treating early-stage hormone receptor (HR)-positive BC in multiple clinical trials. The conventional treatment, tamoxifen, has been proven to lower the risk of contralateral BC and systemic recurrence while lowering the annual death rate from BC by 31%. Since then, various tactics have been demonstrated to improve long-term results [27]. These include aromatase inhibitors (AI), ovarian suppression, and extended AHT beyond five years. The patients need to follow prescribed regimens for the medication to be effective in producing the desired results. This seemingly basic need, however, can be exceptionally challenging for oral medications. Adherence, which is a measure of how closely the patient follows the physician's instructions when taking the medication (daily for AHT), and persistence, which is the total amount of time the patient takes the medication from the time they start taking it until they stop, make up for compliance [19].

Despite the use of AHTs demonstrating survival advantage, a majority of patients stop their medication. While AHT adherence is often excellent in adjuvant trials, it is generally poor in clinical settings, with only around 50% of females completing the recommended five years of AHT. Early AHT cessation and nonadherence are linked to higher BC mortality and recurrence rates [4]. However, in an interesting observation, a recent study noted that adherence to AHT did not influence disease progression and mortality [28]. Adherence to endocrine therapies among BC patients could vary depending on the geographical region and other factors noted from the previous study that reported medication adherence between 52.4% and 84.8% [29].

Factors affecting medication adherence

Older individuals are most affected by cancer diseases. Treatment adherence is paramount to achieve the best results, such as a cure and an improvement in the quality of life. Age-related comorbidities and sensory and cognitive deficits can all impact adherence [30]. Patients might not be adequately informed or knowledgeable about their therapeutic regimen. In patients who received drug information and adherence counseling at the onset of their illness but failed to follow up, this could be due to their forgetfulness. It may be challenging to modify the information given if the patient is very ill during counseling, and misconceptions may happen [31].

Patients frequently change their dosages following their comprehension and may not be aware of the importance of taking their medications exactly as directed. They might hold false or wrong beliefs regarding drugs. They may be demotivated and believe that they have no control over the sickness. A patient may have trouble understanding written language if it is not written in their mother tongue and if they have poor health literacy, which worsens the adherence issue. Comorbidity may raise the likelihood that patients fail to follow through. To manage various drugs and integrate them into daily life, patient motivation is crucial.

Poor medication adherence is a result of difficult access to healthcare and lengthy wait times [32]. It has been observed that both intentional and unintentional forgetfulness and the cost of drugs were the main reasons for nonadherence in Latino/Hispanic postmenopausal females who were suffering from breast cancer [33]. Orally administered anticancer drugs and their adherence are found to be influenced by various factors that include access to drug-related information, shared decisions wherein both the patient and the physicians take responsibility for the consequences, and the role played by the nurse in assessing the factors that interfere with the adherence [34].

In a study from China, Wang et al. noted that medication adherence was greatly affected by the lack of knowledge of medication safety among the patients. It has been identified that older people with comorbidities and those from poor socioeconomic regions are prone to nonadherence [35]. Adherence to oral anticancer medication was strongly associated with frequent visits to the healthcare facility, especially among liver and lung cancer patients [36]. Three main factors that contributed to medication nonadherence among lung cancer patients included intermittent medication, over-adherence, and forgetfulness. However, other factors such as personal, interpersonal, sociocultural, and structural forces were found to be responsible for nonadherence [37]. The lack of low-income subsidy, previous intravenous therapy, healthcare-associated costs, the lack of medical insurance, and out-of-pocket spending have a significant influence on oral anticancer treatment adherence [38].

Barriers to medication adherence

Medication adherence might be impacted by stress and hopelessness. Patients who use injectable medications may find them uncomfortable and fear doing so may harm their bodies. The physical constraints of patients may also provide challenges while administering the medication, which may call for steady hands or clear vision [39]. A patient may have trouble understanding written language if it is not written in their mother tongue, and low health literacy worsens the adherence issue. BC poses a greater risk to life than other health conditions such as diabetes or hypertension. This is evident by fatigue and extreme exhaustion brought on by the cancer illness itself, which could influence medication adherence [40].

**Figure 2:**
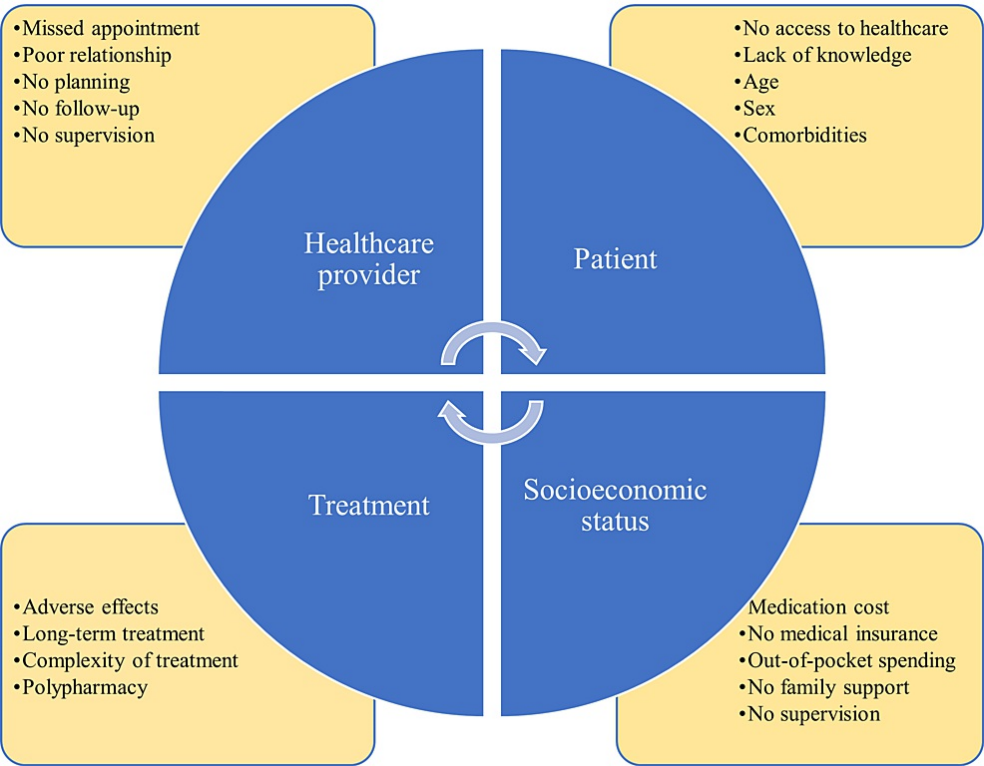
Barriers to anticancer medication adherence Image credits: Venkataramana Kandi

Treatment issues might result from fragmented care between several prescribers, a breakdown in communication between a general practitioner and a community pharmacist, and ineffective coordination between primary and secondary care [41]. In a recent study carried out among lung cancer patients, access to medication and healthcare was cited as the predominant reason for nonadherence [42]. In a recent study that assessed medication adherence among patients with hematologic malignancies, it was observed that only 25% of patients adhered to medications, and many patients discontinued therapy for more than 30 days. Several barriers to medication were identified, including provider, patient, treatment, and socioeconomic factors [43]. The barriers to medication adherence are depicted in Figure 2.

Facilitators of medication adherence

Adherence requires a thorough grasp of the condition, its treatment, and how drugs improve quality of life. The capacity to incorporate drugs into daily living enhances adherence to chronic illnesses that can be self-managed. Low toxicity, mild side effects, and the oral delivery of the drug appear to encourage medication compliance [44]. The important facilitator for medication adherence is patient motivation. If a patient is aware of the advantages of the drug and its need, their motivation will increase. Support from friends, family, and co-workers encourages medication adherence. It might be necessary to disclose the sickness, which the patient might find terrifying [45].

Medication adherence requires a good relationship between patient and physician that could be built on trust, cooperation, and respect. Patients must have easy access to healthcare and sufficient talking time. When dealing with real-world issues in daily life while fighting breast cancer, self-efficacy is a crucial skill [46]. The likelihood of improved adherence is raised if the patient assumes responsibility for self-managing the medication and is knowledgeable about changing medicines if the disease worsens. Good communication and adequate information are the most critical factors in facilitating better medication adherence among breast cancer patients [47]. Recently, a web-based application called BreCanSurvPred was suggested as a method that oncologists could use to improve communications with patients and advise them about the benefits of adherence and hazards of nonadherence to treatment [48].

**Figure 3:**
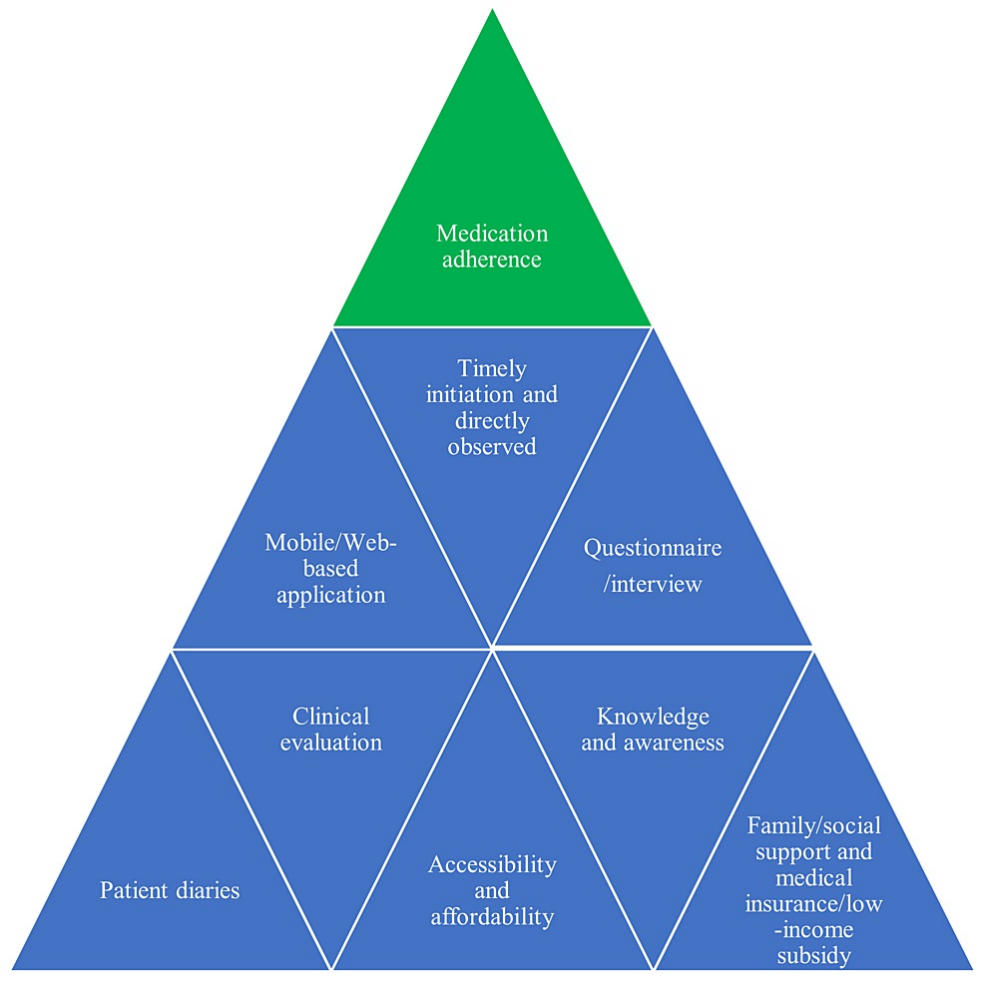
Factors that contribute to improved medication adherence among cancer patients Image credits: Venkataramana Kandi

Improvement in self-medication adherence was noted among the patients who were advised to use a mobile-based application [49]. The availability of generic aromatase inhibitor drugs caused an increase in drug switching and facilitated adherence among older females with breast cancer [50]. Despite the satisfactory levels of medication adherence, long-term adherence may be improved with the interventions of physicians and pharmacists and by using an application that allows patients to adhere to the regimen and contributes to reducing drug wastage [51]. It has recently been suggested that a relationship between the provider, the specialist, and the patient be developed based on the type of cancer to achieve effective and long-term medication adherence [52]. The factors that contribute to improved treatment adherence are depicted in Figure 3.

## Conclusions

Adverse health outcomes are linked to nonadherence to cancer medication. Medication adherence should be approached with an increased patient-centered approach. Cancer therapy is a complex issue, and it requires a multifaceted approach wherein the family and healthcare provider play a crucial role. The financial burden of medical care increases for patients and society at large if patients do not receive the anticipated health benefits and low-income subsidies. Nonadherence to cancer medications could result in unfavorable clinical outcomes and cause increased morbidity and mortality. Moreover, nonadherence to cancer treatment causes tremendous financial, physical, and psychosocial burden on the patients. The timely initiation of anticancer medication is essential for medication adherence and better clinical outcomes.
